# Missed Appointments and the Potential Correlation between Personal Characteristics, Personality, and Familial Characteristics and Missed Appointments for Adults with Diabetes Mellitus at the Primary Care Unit of Khon Kaen Province

**DOI:** 10.3390/healthcare12191992

**Published:** 2024-10-06

**Authors:** Natsuda Sae-Ueng, Varisara Luvira

**Affiliations:** Department of Community, Family and Occupational Medicine, Faculty of Medicine, Khon Kaen University, Khon Kaen 40002, Thailand; natsuda_s@kkumail.com

**Keywords:** appointment, diabetes mellitus, family, personality

## Abstract

Background/Objectives: Regular follow-up treatment is important for the management of diabetes and to reduce the risk of complications. In this study, we aimed to evaluate the proportion of adult diabetic patients who miss appointments, in addition to the potential correlation between personal characteristics, personality, and the context of family structure and characteristics and missed appointments by adult patients with type 2 diabetes. Methods: This study was a cross-sectional descriptive study. The data were gathered through self-administered questionnaires and the patient medical records of 106 individuals who received healthcare services at the Khon Kaen Province primary care unit. Data were gathered from 1 November 2023 to 28 December 2023. Adjusted odds ratios (aORs) and Chi-Square statistics were used to evaluate the relationships with multivariate analyses via multinomial logistic regression and the Kruskal–Wallis test. Results: The majority of patients in the sample, 39.62%, regularly missed appointments. There was a significant association between occasionally missed appointments and middle adulthood (*p*-value 0.013) and regular exercise (*p*-value 0.025). A moderate level of the agreeableness personality trait showed a significant association with missed appointments (*p*-value 0.042). Conclusions: It is important to have a comprehensive understanding of the patient’s personality and family characteristics to effectively plan their healthcare and provide optimal support for diabetes treatment.

## 1. Introduction

Diabetes mellitus (DM) is a chronic, non-communicable disease and significant public health concern, impacting the well-being of individuals, families, and the nation. According to the International Diabetes Federation, statistics from 2021 reveal that more than 537 million individuals suffer from diabetes worldwide. Most adult diabetic patients reside in low- and middle-income nations, accounting for almost 75% of the total number of cases [1]. At present, the number of adults diagnosed with diabetes in Thailand between the ages of 20 and 79 amounts to more than 5 million, and this number is steadily rising [2]. DM is anticipated to pose a significant challenge in the future, both globally and in Thailand.

Approximately 90–95% of all diabetic patients are affected by type 2 diabetes. Several factors contribute to its etiology. It is commonly diagnosed in individuals who have a higher body weight or are classified as obese. Insulin resistance can be caused by carrying excess weight [3]. The disease is characterized by gradual progression. Initially, there are typically no indications or manifestations of the disease. The prolonged elevation of blood sugar levels leads to the manifestation of diabetes symptoms. Sufferers can encounter dangerous symptoms such as high or low blood sugar levels. Chronic complications that impact large arteries can also arise, including stroke, coronary heart disease, and peripheral artery disease, and affect small arteries, leading to potential complications in the eyes, kidneys, and feet. When these complications arise, such as chronic wounds on the feet that necessitate extended hospital treatment, the duration of treatment can vary significantly depending on individual circumstances. As a result of these complications, many individuals unfortunately require leg amputation, suffer from vision loss, or require dialysis as a result of chronic kidney failure, with patients incurring significant treatment expenses [4]. Diabetes not only impacts the well-being of patients, but also their families, both physically and mentally. Moreover, the disease has substantial economic and social implications, both directly and indirectly. Due to expenses incurred from treatment costs, those unable to carry out daily activities independently often find themselves relying on their family or community for support. This situation can lead to a greater sense of dependency and a loss of income, both from the individual’s inability to work due to disability and from caregivers needing to take time off from their jobs to provide care. As such, the disease impacts the well-being of both patients and their loved ones. Adults with diabetes are unable to control their blood sugar levels and face a challenge in maintaining their sugar levels at the level desired in their daily lives [5].

In previous studies, the frequency of treatment noncompliance in diabetes patients was is noted as being extremely high [6]. In a study, poor glycemic control and suboptimal diabetes self-management practices were associated with frequently missed appointments [7]. Furthermore, a diabetes patient’s failure to attend a doctor’s appointment can result in hospitalization or emergency department admission at a later date [8,9]. It has been reported that diabetic patients who neglect to attend appointments experience an increase in micro- and macrovascular complications, suboptimal HbA1C levels, and minimal medical supervision, as well as a higher incidence of morbidity [10,11,12]. In a study, factors associated with missed appointments were classified into categories including patient characteristics, healthcare system and provider factors, and interpersonal factors; however, the results showed inconsistency. The most frequently discussed category is patient characteristics [13]. In some studies, a positive relationship has been discovered between personal characteristics such as gender, age, work-related factors, economic status, duration of disease, and missed appointments [14,15,16,17]. Furthermore, it was found in another study that familial characteristics also have an impact on missed appointments [18].

Personality is a unique characteristic that distinguishes each individual in terms of their thoughts, feelings, and behaviors. It can be classified into five categories: extraversion, agreeableness, conscientiousness, neuroticism, and openness to experience. [19,20]. The results of previous research have demonstrated that personality traits are associated with self-care in individuals with diabetes. In one study, an improvement in foot care compliance was associated with traits such as conscientiousness, openness, and agreeableness. Openness was also associated with improved overall self-care behaviors, conscientiousness was correlated with reduced smoking, and agreeableness was associated with improved medication adherence. Conversely, extraversion and neuroticism were associated with reduced medication adherence, with neuroticism also being associated with lower overall self-care behaviors [21]. However, thus far, no studies have identified the relationship between missed appointments and personality traits.

Primary care units provide treatment close to patients’ homes, with the expectation that patients will receive quality follow-up care. This is particularly important for individuals with chronic conditions who have not yet experienced severe complications in order to prevent the occurrence of such complications in the future. Despite this, research has shown that some patients continue to miss appointments at their local primary care unit [22]. In light of this evidence, the authors of this paper are interested in investigating the proportion of adult diabetic patients who miss appointments, in addition to the potential correlation between personal characteristics, personality, and the context of family structure and characteristics and missed appointments by adults with type 2 diabetes. The findings of this study have the potential to inform the improvement of the care and appointment system for adult diabetic patients seeking services at primary care units.

## 2. Materials and Methods

### 2.1. Study Design and Participants

This study was a cross-sectional, descriptive study. The inclusion criteria were diabetic patients who were at least 20 years of age [23] and willing to participate in the study. In addition, the inclusion criteria included self-sufficient individuals with full awareness who were not suffering from any psychiatric illnesses. In addition, diabetes treatment must have been administered to patients in the primary care unit of Khon Kaen Province for a minimum of one year. The exclusion criteria included individuals with acute illnesses, such as conditions necessitating immediate medical attention. The study population consisted of 146 adults with type 2 diabetes who were undergoing treatment at the primary care unit of Khon Kaen Province. We used the WinPepi program to calculate the sample size. The confidence level was set at 95%, the acceptable difference was 0.05, the assumed proportion was 0.5320, and the population size was 146 individuals. The total sample size calculated was 106 cases. Sampling was conducted through the systematic sampling of the diabetic patient registry. The patients’ ages were arranged in numerical order, and the appropriate interval was calculated as 146/106 = 1.38. We established a random start value of 1 and incremented the following sequence by 1.38 in a continuous manner until the target sample size of 106 individuals was achieved (rounded up during the decision stage using a criterion of 0.5 or above). Each member of the study population had an equal probability of being chosen just once.

### 2.2. Questionnaire

Data were collected from patient medical records and a self-administered questionnaire from 1 November 2023, to 28 December 2023. The main researcher developed a questionnaire that was administered to three experts in order to verify the content’s accuracy and comprehensiveness. Thereafter, the researcher enhanced and revised the questionnaire to ensure that it was more comprehensive, resulting in a content validity value of 0.9. Subsequently, the questionnaire was administered to a non-sample population of 30 individuals to evaluate the coefficient of reliability (Cronbach’s alpha coefficient) before actual practice. The coefficient of reliability exceeded 0.7. The values for extraversion, agreeableness, conscientiousness, neuroticism, and openness to experience were as follows: 0.776, 0.756, 0.729, 0.806, and 0.704, respectively.

The research questionnaire was developed by examining the content of theories, concepts, and research related to personal characteristics, familial characteristics, and diabetes. The questionnaire was divided into four sections.

Part 1: Personal information included gender, age, marital status, educational level, occupation, and underlying disease.

Part 2: Five components of personality characteristics. The main researcher developed a questionnaire consisting of 24 items to assess the five components of personality. These items were derived from a review of the NEO Five-Factor Inventory [20]. The concepts were transformed into questions suitable for Thai society’s context and the individuals’ capabilities. The Likert scale format was used to evaluate the behavior level score at four levels: perform every time, practice often, rarely performed, and never practiced. Each question comprised an answer from which to choose, with a rating of 4, 3, 2, or 1 point. For data analysis, the researcher analyzed the scores from the above determination and interpreted the mean using the following formula: Class Interval = Range/Number of Classes. Subsequently, the following criteria were established for calculating the average of the questionnaire’s evaluation of the five personality components (Big Five): a high-level personality trait was indicated by an average score of 3.01–4.00; a moderate-level personality trait was indicated by an average score of 2.01–3.00; and an average of 1.00–2.00 indicated low-level personality traits.

Part 3: Details regarding family structure and characteristics, such as the number of family members, and the roles and responsibilities of the family.

Part 4: Health behaviors encompassing exercise preferences, feelings of boredom or lack of motivation, and adherence to medication regimens.

In parts 1–3, the researcher gathered data directly from patients by utilizing a responder-type questionnaire. In part 4, the researcher gathered data from patient medical records (secondary data) to ensure the utmost accuracy in the research findings.

### 2.3. Operational Definition

Type 2 diabetes patients are individuals who have been diagnosed with the condition by a physician and are currently receiving treatment that includes behavior modification, blood-sugar-lowering medications, and insulin injections. The diagnostic criteria for diabetes depend on blood test results obtained through one of the following methods:

1. Fasting plasma glucose ≥ 126 mg/dL;
2. Random plasma glucose ≥ 200 mg/dL;
3. Oral glucose tolerance test ≥ 200 mg/dL;
4. Hemoglobin A1c ≥ 6.5%.

The term “adult” in this study refers to those who are 20 years of age or older, with the participants’ ages divided into three stages: early adulthood (aged 20 to 35), middle adulthood (aged 36 to 64), and late adulthood (aged 65 and older) [24].

A missed appointment refers to a situation where the patient fails to attend a scheduled visit with their doctor. Next, “occasionally missed appointment” refers to the act of not attending a scheduled appointment on 10% to 30% of occasions and “regularly missed appointment” refers to the act of not attending a scheduled appointment on more than 30% of occasions.

The five components of personality were as follows:

1. Extraversion is a trait that is often associated with individuals who enjoy socializing, meeting new people, and engaging in conversations. They tend to have an open-minded perspective and maintain positive and enthusiastic behavior.
2. Agreeableness is a trait that is often associated with individuals who tend to be accommodating, cooperative, and compatible in their interactions with others.
3. Conscientiousness is a trait that is often associated with individuals who demonstrate a strong sense of responsibility, possess a disciplined work ethic, set clear objectives, and prioritize careful planning.
4. Neuroticism is a trait that is often associated with individuals who experience a range of negative emotions and emotional instability, including anxiety, fear, sadness, and anger.
5. Openness to experience is a trait that is associated with individuals who are often imaginative, enjoy trying new things, seek out a variety of activities, and are receptive to a range of emotions.

An extended family is a familial unit that includes the nuclear family as well as additional relatives such as grandparents, aunts, and uncles.

Other families can be classified as either one-generation families, which consist of a husband and wife, or two-generation families, which consist of a father, mother, and children. There are also families with special characteristics, such as skipped generation families or families living alone.

The head of the family is the individual who has primary responsibility for all family members and plays a crucial role in resolving family issues.

### 2.4. Data Collection

A letter was sent by the researcher from the head of the Department of Community, Family Medicine, and Occupational Medicine to the hospital director requesting permission from the head of the primary care unit and doctors who regularly treat patients at the unit to collect data from medical records and questionnaires after explaining the details of the study and obtaining consent from the volunteers. The main researcher provided an in-person explanation of the research project at the primary care unit during the data collection period from 1 November 2023, to 28 December 2023, either while the subjects were awaiting examination or after the examination. Following this stage, the research assistant was responsible for obtaining the consent of volunteers who visit the primary care unit for an examination before beginning data collection for the research project.

The data collection process was divided into two stages.

1. Utilization of a self-administered questionnaire: The duration of data collection was approximately 30 min.
  1.1 Information on the research objectives and procedures was provided to the research participants, and they were requested to provide their consent.
  1.2 The research participants were requested to complete a self-administered questionnaire in parts 1–3.
  1.3 In instances where the volunteer was unable to read, the research assistant would read the questions to the volunteer and record their response verbatim.

2. The researcher gathered data in part 4 of the questionnaire from the patient’s medical records.

The researcher gathered all of the data acquired from the participants, examined the correctness and completeness of the data, and then coded the data for data import. The coded data were recorded twice (double entry) by two recorders—the researcher and the research assistant—and then evaluated for consistency in data recording using the statistical software SPSS. If it was determined that the data did not match, the data were checked with the original copy of the data and corrected before being analyzed.

### 2.5. Statistical Analysis

The data collected from the sample were analyzed using the statistical software SPSS (version 28) for comprehensive data analysis. The participants’ characteristics were summarized utilizing descriptive statistics. Frequency and percentages were utilized for categorical variables. To analyze the correlation between personal factors and missed appointments, we conducted multivariate analyses using multinomial logistic regression to estimate the odds ratio and its 95% confidence interval. In the multivariate analysis, we accounted for the influence of confounding variables, which were chosen based on the following criteria: (i) variables that exhibited a *p*-value of less than 0.2 in the crude analysis and (ii) variables identified in prior studies as having an impact on missed appointments. Additionally, to examine the correlation between personality and missing appointments, we employed a univariate analysis utilizing Chi-Square statistics and the Kruskal–Wallis test.

### 2.6. Ethical Approval

This study received approval from the Khon Kaen University Ethics Committee for Human Research and followed the Declaration of Helsinki and ICH Good Clinical Practice Guidelines (No. HE661412). We obtained additional informed consent from all individual participants whose identifying information was included in this article.

## 3. Results

### 3.1. Personal Information and Health Behavior

In this study, data were gathered from a sample of 106 individuals, with the majority (69) being females (65.09%). The median age of the population was 65 years, with an interquartile range of 58–75. A total of 61 individuals (57.54%) were married, the majority of individuals, 89 (83.96%), had completed schooling below secondary school level, and 55 individuals (51.89%) were unemployed. The most common comorbidities included high blood pressure in 68 people (64.15%) and dyslipidemia in 53 people (50.00%) (Table 1).

### 3.2. Correlation between Personal Characteristics and Missed Appointments

According to our results, 34 individuals with diabetes did not miss any appointments (32.08%), 30 individuals missed an appointment occasionally (28.30%), and 42 individuals missed an appointment regularly (39.62%). There was a significant association between occasionally missed appointments and middle adulthood (*p*-value 0.013) and regular exercise (*p*-value 0.025) (Table 2).

### 3.3. Correlation between Personality and Missed Appointments

The results revealed no significant differences in the median levels of the five personality types among diabetic patients who attended all appointments, occasionally missed appointments, and regularly missed appointments (Table 3).

In this study, a significant correlation was discovered between the agreeableness personality trait and the occurrence of missed appointments. Patients who exhibited a moderate level of the agreeableness personality trait were found to have a considerably greater frequency of missed appointments (Table 4).

### 3.4. Correlation between Familial Characteristics and Missed Appointments

No significant statistical relationship between familial characteristics and missed appointments was found in this study (Table 5).

## 4. Discussion

Our study results show that the majority of diabetic patients (67.92%) in our study group failed to attend their appointments on time, with the highest proportion reporting regularly missed appointments (39.62%). The prevalence of missed appointments was significantly higher in this study than that reported in previous studies [9,18,25]. Interestingly, during this study, we examined primary care settings close to the patients’ place of residence; despite this, a significant number of missed appointments were still reported. This finding may be attributed to the fact that nearly half of the participants in the study were employed and had work commitments, in addition to the finding that younger patients were more likely to miss appointments [18,22]. In general, receiving medical treatment during work hours is inconvenient. In addition, some elderly individuals require assistance from a caregiver in order to collect their medication. Based on the time of the appointment, the caregiver may be unable to collect the medication due to their work commitments. Alternatively, the patient may be responsible for household duties, such as childcare. Thus, it is sometimes inconvenient for individuals to collect medication during scheduled appointments [18]. Notably, the primary care unit is conveniently located near to the patient’s home, allowing them to visit the doctor at a time that is convenient for them. Thailand’s healthcare system works in such a way that individuals can visit a doctor without requiring a prior appointment. Additionally, some individuals may choose to wait until their medication supply has run out before seeking a refill. From the results obtained in this study, it is evident that despite missing their appointments, the participants consistently adhered to their medication regimen. The results of another study revealed that only 2.83 percent of patients failed to take their medication.

The results of this study indicate that regularly missed appointments are not associated with any personal characteristics or health behaviors. However, age in middle adulthood and regular exercise were found to be factors associated with occasionally missed appointments. Based on age, individuals in middle adulthood were more prone to occasional missed appointments, corresponding with the results of a systematic review in which it was found that younger persons had lower attendance rates at medical visits [14]. This finding may be due to the patients being in middle adulthood, a stage characterized by the expectation of multiple responsibilities in life. With responsibilities pertaining to family, career, and society [26], health may not be afforded significant priority. In general, it can be quite inconvenient when a doctor’s appointment to follow up on treatment coincides with work commitments. Individuals in this age group may occasionally miss appointments due to work-related commitments, as they attempt to maintain a professional balance between their health and career responsibilities [18]. Regarding exercise, it is interesting to note that individuals who engage in regular exercise exhibit a correlation with having occasional missed appointments, but no correlation with experiencing routinely missed appointments. This finding contrasts with other research findings that have identified personal characteristics as a major contributor to diabetic patients’ non-attendance at appointments [13]. In one study, consistently failing to attend appointments was found to be associated with poorer glucose control and insufficient self-care practices among individuals with diabetes [7]. Engaging in physical exercise is a crucial aspect of self-care for individuals with diabetes [25]. Therefore, diabetic patients who engage in regular physical activity are individuals who adhere to good self-care practices. However, some individuals in Thailand are hesitant to take medication due to concerns about potential side effects on the kidneys [27]. As a result, a number of patients seek alternative healthcare options to minimize or discontinue their reliance on medication. This patient population may consider exercise as an alternative to medication, as they believe that it can reduce the need for medication. Consequently, such patients occasionally fail to attend appointments. Given that patients may attempt to stop taking their medication or decrease their medication dose while engaging in regular exercise, it is therefore possible that the patient may have some medication remaining on the day of their scheduled doctor’s appointment. As a result, the patient may decide not to attend their appointment. However, a responsible individual would not frequently miss appointments, as they understand the importance of maintaining their health.

According to the results of this study, patients with a moderate level of the agreeableness personality trait were found to have a significantly higher rate of missed appointments. This is the first study to investigate the correlation between personality and missing appointments. People with a personality type characterized by agreeableness often display traits such as being accommodating, cooperative, and compatible in their interactions with others. Previous research has demonstrated that diabetic patients with an agreeable personality type will exhibit improved adherence to medication and foot care [21], which represents one of the self-care practices. However, the results of this study were inconsistent in terms of missed appointments [7], which were associated with self-care practice. It is possible that individuals with an agreeableness personality type exhibit effective self-care practices in the areas of medication adherence and foot care compliance, which are clearly associated with self-care in the direct treatment of diabetes. However, it is not possible to directly compare this personality type in terms of following up on treatment, as it is exclusively associated with self-care practices. Individuals with an agreeable personality type typically exhibit a compliant nature and prioritize the interests of others over their own [20]. As noted above, Thailand’s public health system allows individuals to see a doctor without a prior appointment, particularly for primary care services that are conveniently located near their homes and have low patient volumes. Consequently, this group of individuals may not adhere to scheduled appointments due to engagement in other activities or the belief that visiting the doctor as scheduled is not of great importance. They may also choose to wait until their medication runs out before seeking medical attention.

In this study, we gathered data from diabetic individuals seeking treatment at the primary care unit of Khon Kaen Province. The province is large and has substantial economic expansion in the northeastern area of Thailand. Patients receiving treatment for diabetes at the primary care unit comprise individuals who have not yet experienced significant complications related to their condition. The data could, therefore, potentially reflect the diabetic patient population. The results of this study reveal intriguing preliminary findings on the potential correlation between diabetes patients who do not engage in regular exercise and those with an agreeable personality type and their propensity to miss appointments. Despite the findings being preliminary, the data collected in this research are representative of a broad age range, spanning from early adulthood to old age, resulting in distinct contexts in the lives of individuals in each age group. Moreover, the system for scheduling appointments with physicians in Thailand provides flexibility. Even without scheduling an appointment, it is possible to consult a doctor, particularly in the field of primary care. This practice may vary among other nations. Regarding data collection, the use of self-administered questionnaires in this study may create bias, since individuals may either underreport or overreport certain behaviors, which is a particular issue given the small sample size. Thus, this issue could affect the accuracy of the data, and it is also important to note that the study method used is descriptive, necessitating cautious interpretation of data regarding the correlation between factors. It is therefore necessary to validate the results of this study with a more prospective study in the future.

## 5. Conclusions

The majority of individuals with diabetes consistently fail to attend their appointments. Missed appointments may be associated with personality characteristics that reflect agreeableness, with middle adulthood and regular exercise as additional factors that are associated with occasionally missed appointments. In addition, in this study, no significant correlation was identified between familial characteristics and missed appointments for treatment. Therefore, it is important to recognize and appreciate the influence of personal characteristics and personality traits. This understanding plays an important part in developing the patient’s healthcare plan, including scheduling treatment appointments, to ensure the effective management of diabetes.

## Figures and Tables

**Table 1:** Personal information and health behaviors.

Characteristics	Overall n (%)	Type of Missed Appointments			*p*-Value
		No Missed Appointments n (%) 34 (32.08)	Occasionally Missed Appointments n (%) 30 (28.30)	Regularly Missed Appointments n (%) 42 (39.62)	
Gender					
Male	37 (34.91)	11 (29.73)	12 (32.43)	14 (37.84)	0.784
Female	69 (65.09)	23 (33.33)	18 (26.09)	28 (40.58)	
Age (years)					
36–64	51 (48.11)	12 (23.53)	18 (35.29)	21 (41.18)	0.136
65 and older	55 (51.89)	22 (40.00)	12 (21.82)	21 (38.18)	
Status					
Single	8 (7.55)	2 (25.00)	2 (25.00)	4 (50.00)	0.661
Married	61 (57.54)	19 (31.15)	24 (39.34)	18 (29.51)	
Widower	29 (27.36)	7 (24.14)	11 (37.93)	11 (37.93)	
Divorce/Separated	8 (7.55)	1 (12.50)	2 (25.00)	5 (62.50)	
Education					
Below secondary school level	89 (83.96)	28 (31.46)	27 (30.34)	34 (38.20)	0.642
Secondary school or higher	17 (16.04)	6 (35.29)	3 (17.65)	8 (47.06)	
Career					
Unemployed	55 (51.89)	19 (34.54)	13 (23.64)	23 (41.82)	0.539
Employed	51 (48.11)	15 (29.41)	17 (33.33)	19 (37.26)	
Underlying disease					
Diabetes mellitus	106 (100.00)	34 (32.08)	30 (28.30)	42 (39.62)	
Hypertension	68 (64.15)	23 (33.82)	18 (26.47)	27 (39.71)	0.816
Dyslipidemia	53 (50.00)	15 (28.30)	14 (26.42)	24 (45.28)	0.482
Chronic kidney disease	3 (2.83)	0 (0.00)	1 (33.33)	2 (66.67)	0.452
Stroke	4 (3.77)	1 (25.00)	2 (50.00)	1 (25.00)	0.612
Other	10 (9.43)	4 (40.00)	3 (30.00)	3 (30.00)	0.784
Exercise regularly					
No	26 (24.53)	8 (30.77)	3 (11.54)	15 (57.69)	0.043
Yes	80 (75.47)	26 (32.50)	27 (33.75)	27 (33.75)	
A feeling of boredom or disinterest					
No	82 (77.36)	24 (29.27)	25 (30.49)	33 (40.24)	0.464
Yes	24 (22.64)	10 (41.67)	5 (20.83)	9 (37.50)	
Medication adherence					
No	3 (2.83)	1 (33.34)	1 (33.33)	1 (33.33)	0.970
Yes	103 (97.17)	33 (32.04)	29 (28.15)	41 (39.81)	

**Table 2:** Personal information and health behaviors associated with missed appointments in multivariate logistic regressions.

Types of Missed Appointments	Characteristics	Adjusted OR	95% CI	Exp (B) (*p*-Value)
Occasionally missed appointments	Gender			
	Male	1		
	Female	0.767	0.236–2.491	0.659
	Age (years)			
	36–64	1		
	65 and older	0.176	0.044–0.696	0.013 *
	Education			
	Below secondary school level	1		
	Secondary school or higher	0.227	0.040–1.280	0.093
	Career			
	Unemployed	1		
	Employed	0.028	0.705–1.499	0.885
	Exercise regularly			
	No	1		
	Yes	6.740	1.268–35.812	0.025 *
	A feeling of boredom or disinterest			
	No	1		
	Yes	0.429	0.115–1.596	0.207
	Medication adherence			
	No	1		
	Yes	1.497	0.064–35.096	0.802
Regularly missed appointments	Gender			
	Male	1		
	Female	1.154	0.394–3.380	0.794
	Age (years)			
	36–64	1		
	65 and older	0.402	0.108–1.500	0.175
	Education			
	Below secondary school level	1		
	Secondary school or higher	0.905	0.233–3.523	0.886
	Career			
	Unemployed	1		
	Employed	0.820	0.576–1.166	0.069
	Exercise regularly			
	No	1		
	Yes	0.609	0.189–1.964	0.406
	A feeling of boredom or disinterest			
	No	1		
	Yes	0.663	0.226–1.940	0.453
	Medication adherence			
	No	1		
	Yes	0.780	0.038–15.998	0.872

Multivariable analysis was used to estimate adjusted odds ratios via multinomial logistic regression by adjusting for gender, age, education, career, exercise, feeling of boredom or disinterest, and medication. * Results were considered significant at *p* < 0.05.

**Table 3:** The median level of the five personality types in adults with diabetes mellitus, which includes no missed appointments, occasionally missed appointments, and regularly missed appointments.

Personality Type	Median (IQR)				Kruskal–Wallis Test (*p*-Value)
	Overall	No Missed Appointments	Occasionally Missed Appointments	Regularly Missed Appointments	
Extraversion	3.40 (3.00–3.80)	3.60 (3.20–3.85)	3.20 (3.00–3.65)	3.30 (3.00–3.80)	0.320
Agreeableness	3.75 (3.25–4.00)	3.75 (3.25–4.00)	3.63 (3.25–4.00)	3.50 (3.00–3.75)	0.236
Conscientiousness	3.60 (3.2–3.85)	3.60 (3.40–4.00)	3.50 (3.00–3.85)	3.50 (3.15–3.85)	0.441
Neuroticism	2.00 (1.80–2.40)	2.00 (1.60–2.25)	2.00 (1.80–2.40)	2.10 (1.80–2.45)	0.260
Openness to experience	3.00 (2.60–3.20)	3.00 (2.40–3.20)	3.00 (2.75–3.20)	2.80 (2.40–3.20)	0.379

**Table 4:** The levels of the five personality types in adults with diabetes mellitus, which include no missed appointments, occasionally missed appointments, and regularly missed appointments.

Characteristics	n (%)	No Missed Appointments n (%)	Occasionally Missed Appointments n (%)	Regularly Missed Appointments n (%)	Chi-Square Test (*p*-Value)
Extraversion					
High level	78 (73.58)	28 (35.90)	22 (28.20)	28 (35.90)	
Low–moderate level	28 (26.42)	6 (21.43)	8 (28.57)	14 (50.00)	0.304
Agreeableness					
High level	86 (81.13)	32 (37.21)	24 (27.91)	30 (34.88)	
Moderate level	20 (18.87)	2 (10.00)	6 (30.00)	12 (60.00)	0.042 *
Conscientiousness					
High level	86 (81.13)	32 (37.21)	22 (25.58)	32 (37.21)	
Moderate level	20 (18.87)	2 (10.00)	8 (40.00)	10 (50.00)	0.061
Neuroticism					
High level	3 (2.83)	1 (33.33)	0 (0)	2 (66.67)	
Moderate level	44 (41.51)	13 (29.55)	12 (27.27)	19 (43.18)	0.726
Low level	59 (55.66)	20 (33.90)	18 (30.51)	21 (35.59)	
Openness to experience					
High level	37 (34.91)	12 (32.43)	12 (32.43)	13 (35.14)	
Moderate level	62 (58.49)	19 (30.64)	17 (27.42)	26 (41.94)	0.860
Low level	7 (6.60)	3 (42.86)	1 (14.28)	3 (42.86)	

* Results were considered significant at *p* < 0.05.

**Table 5:** The correlation between familial characteristics and regularly missed appointments.

Familial Characteristics	Overall n (%)	No Missed Appointments	Occasionally Missed Appointments	Regularly Missed Appointments	Chi-Square Test (*p*-Value)
Family structure (n, %)					
Extended family	49 (46.23)	15 (30.61)	12 (24.49)	22 (44.90)	0.557
Other families	57 (53.77)	19 (33.33)	18 (31.58)	20 (35.09)	
Number of family members (Median, IQR)	4 (3–5)	4 (3–5)	4 (2.75–5.00)	4 (2.75–6.00)	0.640
Family role (n, %)					
Family leader	59 (55.66)	17 (28.81)	18 (30.51)	24 (40.68)	0.702
Family member	47 (44.34)	17 (36.17)	12 (25.53)	18 (38.30)	

## Data Availability

The datasets used and/or analyzed during the current study are available from the corresponding author upon reasonable request.
